# Involvement of Gut Microbiota, Microbial Metabolites and Interaction with Polyphenol in Host Immunometabolism

**DOI:** 10.3390/nu12103054

**Published:** 2020-10-06

**Authors:** Andy W.C. Man, Yawen Zhou, Ning Xia, Huige Li

**Affiliations:** Department of Pharmacology, Johannes Gutenberg University Medical Center, Langenbeckstr. 1, 55131 Mainz, Germany; wingcman@uni-mainz.de (A.W.C.M.); yawezhou@uni-mainz.de (Y.Z.); xianing@uni-mainz.de (N.X.)

**Keywords:** inflammation, gut microbiota, polyphenol, metabolic diseases, metabolites

## Abstract

Immunological and metabolic processes are inextricably linked and important for maintaining tissue and organismal health. Manipulation of cellular metabolism could be beneficial to immunity and prevent metabolic and degenerative diseases including obesity, diabetes, and cancer. Maintenance of a normal metabolism depends on symbiotic consortium of gut microbes. Gut microbiota contributes to certain xenobiotic metabolisms and bioactive metabolites production. Gut microbiota-derived metabolites have been shown to be involved in inflammatory activation of macrophages and contribute to metabolic diseases. Recent studies have focused on how nutrients affect immunometabolism. Polyphenols, the secondary metabolites of plants, are presented in many foods and beverages. Several studies have demonstrated the antioxidant and anti-inflammatory properties of polyphenols. Many clinical trials and epidemiological studies have also shown that long-term consumption of polyphenol-rich diet protects against chronic metabolic diseases. It is known that polyphenols can modulate the composition of core gut microbiota and interact with the immunometabolism. In the present article, we review the mechanisms of gut microbiota and its metabolites on immunometabolism, summarize recent findings on how the interaction between microbiota and polyphenol modulates host immunometabolism, and discuss future research directions.

## 1. Introduction

The worldwide prevalence of obesity has emerged as a major cause of immunometabolism diseases including diabetes and cardiovascular diseases [1]. Immunometabolism has recently been described as the interface of the immune system and metabolism [2,3,4]. Indeed, chronic non-communicable diseases such as obesity, type 2 diabetes, and cardiovascular disease are recognized as the disruption of the interaction between the immune system and metabolism [5]. The increasing prevalence of immunometabolic diseases and the complicated connections between metabolic dysregulation and inflammation underline the need to reveal the metabolic programming of immune cells. The emerging field of immunometabolism has highlighted the significance of cellular metabolism in the regulation of immune cell activity. Under certain conditions, anabolic and catabolic metabolisms have become associated with pro- and anti-inflammatory immune responses, respectively [3]. Thus, modulation of specific metabolic pathways in immune cells may represent a novel strategy to inhibit inflammation and to promote the anti-inflammatory immune responses.

Gut microbiota is a complex ecosystem, which is composed by numerous species of microorganisms that highly interact with the host. The symbiotic interaction between the host and gut microbiota is important for the host’s metabolism and health [6]. Therefore, gut microbiota has been considered as a virtual endocrine system with their metabolites modulating the host’s metabolic functions. Most of the gut microbiota identified consist of five phyla: *Bacteroidetes, Firmicutes, Actinobacteria, Proteobacteria,* and *Verrucomicrobia.* Their relative abundance and diversity of species are highly variable, and anaerobic *Bacteroidetes* and *Firmicutes* usually occupy more than 90% of the total gut microbiota population [7,8]. The composition of the gut microbiota can be affected by the host genome and environmental factors, such as usage of antibiotics and probiotics, lifestyle, hygiene status, and diet [8]. Due to the advancement of genome sequencing technologies, bioinformatics becomes an excellent research tool to identify and characterize the composition of microbiota [9]. It is widely accepted that gut microbiota dysbiosis is correlated with metabolic disorders and serious vascular complications [10]. Changes in dietary patterns promote a quick and reversible populational change of dominant gut microbiota [11]. Cardiometabolic diseases, such as obesity and diabetes, have been described as the result of an intricate crosstalk between individual genotypes, aging, environmental factors, dietary pattern, as well as the gut microbiota [12]. 

Polyphenols are water-soluble phenolic secondary metabolites of plants, and are categorized into several types including flavonoids, phenolic acids, and phenolic amides according to their chemical structures [13]. Polyphenols can be found largely in fruits and vegetables, while for humans, common sources of dietary polyphenol also include beverages and herbal products [14]. Polyphenols possess anti-oxidant and anti-inflammatory properties, while they are also important immunonutrients that have been extensively studied in the context of immune diseases [15,16,17,18]. However, polyphenols have relatively low bioavailability in our body, less than 10% of total polyphenol intake is absorbed in small intestines. The unabsorbed polyphenol may accumulate in large intestines and be metabolized by the gut microbiota into smaller, low molecular weight phenolic metabolites and absorbed into the body [19,20]. In addition, many studies support the theory that polyphenols with poor bioavailability possibly act primarily through the gut microbiota remodeling, which affects the microbial composition and function [21]. The majority of polyphenol researches have focused on their antimicrobial activity, but the newly emerging concept of polyphenols as potential prebiotics to shape the gut microbiota composition is concerned [22]. In this review, we will update different studies exploring the effect of gut microbiota and its metabolites on host immunometabolism. We will also describe why and how the interplay between polyphenol and gut microbiota helps to modulate immunometabolism and prevent immunometabolic complications.

## 2. Role of Gut Microbiota in Host Energy Metabolism and Immune Functions

Gut microbiota plays a central role in maintaining metabolic and immunological functions of host tissue and organs. In relation to the energy metabolism of the host, gut microbiota is involved in various metabolic functions, including enzymatic digestion, fermentation and absorption of complex dietary carbohydrates and proteins, and providing essential vitamins and amino acids for the host [23,24]. Increasing evidence from preclinical and clinical studies has highlighted that gut microbiota can modify immunometabolism by modulating epithelial and immune cells, which results in immune-inflammatory responses that favor the progression of diabetes and its complication [25].

Gut microbiota dysbiosis refers to the disruption of the dynamic interaction between the host and the microbial communities, as well as a bacterial imbalance between the ratio of aerobic and facultative anaerobic bacteria [26]. Many diseases, including inflammatory diseases, metabolic disorders, obesity, diabetes, and cardiovascular diseases, have been associated with specific bacterial dysbiosis [27]. Changes in the diversity and population of microbiota species, for example, the reduction of beneficial and anti-inflammatory species (including *Faecalibacterium prausnitzii* and *Akkermansia muciniphila)* or the colonization of proinflammatory bacteria (including *Bacteroides* and *Ruminococcus gnavus)* can facilitate the pathogenesis and chronicity of immunometabolic diseases [27,28]. Currently, *Bacteroides, Lactobacillus*, *Eubacterium,* and *Roseburia* are beneficial genera of bacteria identified to be involved in anti-inflammatory and antioxidant processes [29]. Moreover, *Lactobacillus* can use tryptophan as an energy source and generate ligands of the aryl hydrocarbon receptor (AhR), which is involved in the organogenesis of intestinal lymphoid follicles (ILFs) and affect the secretion of anti-microbial peptides [30]. On the other hand, the Firmicutes to Bacteroidetes ratio is found significantly increased in obese mice [31]. Gut microbiota dysbiosis may also trigger the breakdown of gut barrier integrity which leads to local and systemic inflammation [26,32]. Rupture of the gut barrier integrity facilitates the translocation of bacteria fragments, including lipopolysaccharide (LPS) and peptidoglycan (PG), to the bloodstream. The translocation of bacterial components and other microbial-derived metabolites through the intestinal barrier to the systemic circulation can affect the inflammatory and oxidative state, as well as the cellular metabolism and immunity [11,33].

The concept that nutrients and gut microbiota have important roles in modulating insulin resistance and cellular metabolisms has been demonstrated systemically in animal models and in humans [2]. Indeed, gut microbiota transplantation from lean donors into subjects with metabolic disease can ameliorate insulin sensitivity, suggesting that microbiota and host metabolism are intrinsically linked [33]. By far, at least three major mechanisms have been identified as triggers of obesity-associated metabolic inflammation including endoplasmic reticulum (ER) stress, toll-like receptor 4 (TLR4) activation, and changes in gut microbiota [34]. Indeed, the innate immune component TLR4 has been identified as a receptor for saturated and polyunsaturated fatty acids [35], and TLR signaling is involved in metabolic controls [36]. Moreover, TLR4 can also be activated in response to the LPS produced by Gram-negative bacteria [37]. The microbe-associated molecular pattern (MAMP) can provoke pro-inflammatory responses by activating TLR4, which in turn affect glucose metabolism and insulin signaling [38]. In addition, these pro-inflammatory responses can further stimulate the production of advanced glycation end products (AGEs) and other oxidative pathways, which are also involved in the immunometabolic impairment. Current researches support TLR4 as an important link among gut microbiota and immunometabolism.

Currently, gnotobiotic or germ-free mice are common animal models to study the effects of gut microbiota in host immunometabolism. Indeed, the significance of gut microbiota in modulating host metabolism is suggested when the reduced adiposity of germ-free mice can be reversed by colonization with a normal gut microbiota [39]. In addition, transplantation of microbiota from obese mice to either germ-free or antibiotics-treated mice induces greater weight gain than those receiving from lean mice [40,41]. Moreover, germ-free mice are more resistant to high-fat diet (HFD)-induced body weight gain compared to conventionally raised mice [42]. On the other hand, gnotobiotic mice show underdeveloped lymphoid tissues, impaired T and B cell functions, and decreased number of CD4^+^ T cells and antibody production [43]. Moreover, there is evidence showing that T helper 17 (Th17) and regulatory T cells are less efficient in germ-free mice compared to that of control during infection [44]. Colonization of germ-free mice with specific bacterial species can restore immunological functions [45,46]. However, the results from these germ-free mice studies may lack clinical relevance, as these mice are artificially maintained and lack early exposure to normal flora. The use of antibiotics treatment [47] or humanized animals (germ-free mice transplanted with human fecal microbiota) [48] may be a better model to investigate the interaction between microbiota and polyphenols in immunometabolic disease.

Furthermore, type 1 diabetes is long considered as an autoimmune disease, while it has been recently proposed that the crosstalk between gut microbiota and host immunometabolism may be a common molecular basis of both type 1 and type 2 diabetes [49]. In patients with type 1 diabetes, differences in intestinal microbiota composition are observed, suggesting the potential involvement of the gut microbiota in the etiology of type 1 diabetes [50,51,52]. In addition, a low gut microbe diversity has been linked to type 1 diabetes and β-cell autoimmunity [53]. During infancy, the presence of commensal intestinal microbiota is critical for various physiologic processes including stimulation of various arms of the innate and adaptive immune systems [54]. A randomized, double-blind, controlled intervention trial has also shown that highly hydrolyzed casein can reduce the risk of type 1 diabetes in genetically predisposed children and is associated with the change in gut microbiota composition influencing the development of the immune system [55]. This suggests the significant role of gut microbiota in modulating the host metabolism and immunity, while the symbiosis between the host and gut microbiota is developed from birth and is critical for the host health. Therefore, shaping the gut microbiota can be a promising strategy to prevent the pathogenesis of immunometabolic diseases.

Indeed, the gut microbiota produces extremely diverse metabolites from the anaerobic fermentation of dietary components that reach the colon, as well as endogenous compounds that are generated by both the host and microorganisms [56]. Many of these metabolites are produced by the gut bacteria to inhibit the growth of their competitors, which are considered as a biological strategy for maintenance of diversity of commensal species, and elimination of pathogenic bacteria [57]. In addition, these gut microbiota metabolites are involved in various important physiological processes, including host energy metabolism and immunity. To date, thousands of microbial metabolites with known and unknown functions have been identified as components of the human metabolome [58,59]. Recent studies have been focused on uncovering the major role of bacterial metabolites in the regulation of host immunometabolism. Gut microbes and their metabolites are necessary for the host immune system to distinguish self and non-self at early life, as well as to activate the innate lymphoid cells, natural killer cells, cytotoxic, noncytotoxic, and helper lymphoid cells [60,61]. Here, we summarize the major findings on the effects of gut microbiota metabolites on the host immunometabolism. The growing understanding of the detailed mechanisms on how microbiota regulates host immunometabolism provides new opportunities to interfere with and protect against important pathogenesis.

### 2.1. Short-Chain Fatty Acids

SCFAs are a group of the most important of gut microbiota metabolites fermented from resistant starch or dietary fiber [62]. The predominant bacteria known to produce SCFAs are *Akkermansia muciniphilia, Prevotella* spp., *Ruminococus* spp., *Coprococcus* spp., *Faecalibacterium prausnitzii, Eubacterium rectale*, and *Roseburia* spp. [63]. The major SCFAs include acetate, propionate, and butyrate. SCFAs are transported from the intestinal lumen into the blood circulation of the host and to the organs where they act as substrates or signal molecules. SCFAs are known to modulate multiple metabolism, inflammation, hormone production, lipogenesis, and gut homeostasis via binding to their receptors, which include G protein-coupled receptor 41 (GPR41), GPR43, GPR109A, and vascular olfactory receptor 78 (Olfr78) [64,65]. SCFAs are generally considered to have beneficial effects on host health.

Acetate and propionate are substrates to facilitate ATP production in muscles and liver [66]. Apart from that, SCFAs have been shown to increase the AMP-activated protein kinase (AMPK) activity in muscles and liver, while the activation of AMPK triggers the upregulation of peroxisome proliferator-activated receptor gamma coactivator (PGC-1α) [67]. PGC-1α is an important regulator of cholesterol, lipid, and glucose metabolism, partly by modulating the activity of peroxisome proliferator-activated receptor (PPARα, PPARδ, and PPARγ), liver X receptor (LXR), and farnesoid X receptor (FXR) [68,69]. Moreover, SCFAs have been shown to upregulate PGC-1α and uncoupling protein 1 (UCP1) in brown adipose tissues, and are associated with increased thermogenesis and fatty acid oxidation [67]. On the other hand, SCFAs can inhibit the *de novo* synthesis of fatty acids and lipolysis, which results in a total reduction of plasma level of free fatty acids [70] and a decrease in body weight [71,72]. In addition, SCFAs can trigger the local release of peptide tyrosine-tyrosine (PYY) and glucagon-like peptide-1 (GLP-1) from enteroendocrine L cells [73]. SCFA have been positively associated with elevated plasma levels of PYY and GLP-1 in both humans and mice studies [74,75]. These nutrient-sensing hormones are involved in the modulation of plasma glucose level, lipid metabolism, and fatty acids storage in the liver [76]. PYY is known as a satiety hormone that reduces appetite, while it can also enhance the insulin action on glucose disposal in skeletal muscles and adipose tissues [77,78]. GLP-1 is a key determinant of blood glucose homeostasis, which can promote the secretion of insulin and inhibit the secretion of glucagon by the pancreas [79]. Either administration of SCFAs or intake of dietary fibers increases GLP-1 and PYY plasma levels and glucose uptake by adipose tissue [80,81,82]. In addition, GLP-1 and PYY have receptors expressed in the brain that are involved in the regulation of host energy homeostasis, suggesting the significant contribution of SCFAs in intestine–brain crosstalk [83]. Therefore, high SCFAs levels are negatively correlated to the risk of obesity. Obesity has been associated with a reduced number of butyrate and propionate producing bacterial species in the gut microbiota [84]. Moreover, increased levels of propionate were detected in mice who had gastric bypass and in germ-free recipient mice receiving the gut microbiota from mice who had gastric bypass, whereas the germ-free recipient mice were also protected from diet-induced obesity [85]. In addition, both *in vitro* and *in vivo* experiments suggested that SCFAs increase the production of important adipokine, leptin, from adipose tissue [86,87].

SCFAs have been shown to be involved in immune responses and inflammatory conditions. A metagenomic study suggests that a consortium of lactate- and butyrate-producing gut bacteria can induce a sufficient amount of mucin synthesis to maintain gut integrity and prevent the development of autoimmune diseases including type 1 diabetes [53]. Mice deficient in one of the SCFA receptors, GPR43, show exacerbated inflammation in models of colitis and peripheral inflammation [88]. Moreover, SCFAs have been shown to elevate the number and function of induced regulatory T cells [89]. Propionate is associated with improved glucose homeostasis via improving pancreatic β-cell function [90,91]. Acetate and butyrate are involved in maintaining β-cell function by mediating B cells differentiation and function, enhancing the number and function of regulatory T cells and reducing the population of autoreactive T cells [92]. Butyrate has been shown to upregulate the regulatory T cells, which calm the mucosal immune response, suppress inflammation, and maintain mucosal integrity in the colon [60]. A recent study also suggests that butyrate is able to control the capacity of T cells by differentially regulating Th1 and Th17 cell differentiation and promoting IL-10 production in the induction of colitis [93]. Butyrate also inhibits histone deacetylase 3 (HDAC3) in monocytes during macrophage differentiation and induces metabolic changes that enhance the antimicrobial function of macrophage [94]. Butyrate is also beneficial against inflammation by inhibiting superoxide production and consequent Nod-like receptor pyrin domain 3 (NLRP3) inflammasome formation and activation [95]. On the other hand, butyrate has been shown to upregulate the expression of TLR4 and the phosphorylation of mitogen-activated protein kinases (MAPK) and nuclear factor kappa light chain enhancer of activated B cells (NF-κB) in colon cancer cell *in vitro*, which can further trigger pro-inflammatory response and initiate innate immunity against cancer cells. [96]. In vasculature, SCFAs can inhibit LPS or tumor necrosis factor alpha (TNF-α)-induced endothelial inflammatory responses and excessive vascular cell adhesion molecule 1 (VCAM-1) expression [64]. SCFAs can also induce leptin expression, which has been shown to contribute to both innate and adaptive immune response by stimulating the expression of CD25, CD39, CD69, CD71, and CD121a [97]; proinflammatory cytokines interleukin 6 (IL-6); and TNF-α from adipose tissues [98]. Together these data support that SCFAs are important bacterial messengers that modulate host immunometabolism.

### 2.2. Branched-Chain Amino Acids

BCAAs (including leucine, isoleucine, and valine) are essential amino acids that possess an aliphatic sidechain with a branch. BCAAs cannot be synthesized by humans and must therefore originate from ingested food or gut microbial synthesis. Gut microbiota is known to be involved partly in regulating the biosynthesis, transport, and metabolism of BCAAs [99,100]. *Prevotella copri* and *Bacteroides vulgatus* are known to be the main species that contribute to the increased circulating BCAA levels [101,102], while a potentially causal role of *Bacteroides ovatus* in mediating the biosynthesis of BCAAs in metabolic disorders has also been suggested recently [103]. 

Although BCAAs are now advertised as health supplements to build muscles and reduce exercise fatigue, high levels of BCAA, mainly derived from muscle protein and associated with specific gut microbiota compositions, have recently emerged as contributors of inflammation and may lead to the development of insulin resistance and diabetes [104]. Individuals with insulin resistance have been shown to possess altered microbiota composition and microbiota-derived metabolite profile with higher levels of circulating BCAA [104,105]. In mice, a challenge with *Prevotella copri* led to increased circulating serum levels of BCAAs, insulin resistance, and an aggravation of glucose intolerance [102]. HFD-induced gut dysbiosis is associated with the increased circulating BCAA levels, while oral gavage with *Bacteroides thetaiotaomicron* normalizes serum BCAA levels and alleviates diet-induced body weight gain and adiposity [106].

Mammalian target of rapamycin (mTOR) is a serine/threonine protein kinase present in two signaling complexes, mTORC1 and mTORC2 [107]. mTOR is particularly sensitive to BCAAs, while BCAAs are required for cell proliferation in mTORC1-dependent pathway [108]. High concentration of BCAAs promotes oxidative stress, inflammation and migration of human peripheral blood mononuclear cells via mTORC1 activation [108]. BCAAs trigger ROS formation in peripheral blood mononuclear cells through the activation of nicotinamide adenine dinucleotide phosphate (NADPH) oxidase 2 (NOX2) [108], which is particularly important in immune cells [109]. mTOR signaling is also a central regulator of cellular metabolism and T cell responses [107]. mTORC1 signaling is important for T cell development in the thymus; homeostasis in the periphery; and differentiation into CD4^+^ Th1, Th2, and Th17 cells, as well as cytotoxic CD8^+^ T cells [110,111,112]. mTORC2 is required for Th1 and Th2 cell differentiation [113]. In mice, BCAA-reduced diet decreases the numbers and proliferative capacity of Foxp3^+^ regulatory T cells [114].

Despite several lines of experimental evidence showing the positive association between gut dysbiosis, elevated BCAA levels, and insulin resistance, some observations are controversial. Inhibition of cytosolic branched-chain aminotransferase (BCAT1), the enzyme responsible for the reversible transamination of BCAA, reduces inflammation associated with decreased macrophage infiltration in animal models of collagen-induced arthritis or crescentic glomerulonephritis [115]. Deletion of mitochondrial branched-chain aminotransferase (BCAT2) increases energy expenditure and improved insulin sensitivity [116].

On the other hand, BCATs initiate bacterial branched-chain fatty acid (BCFA) synthesis by converting BCAAs into branched chain α-keto acids [117]. BCFAs are rare in mammalian tissues, while they are essential membrane component of many bacterial species [118]. Dietary BCFA has been shown to increase the expression of the anti-inflammatory cytokine, IL-10, as well as to reduce the incidence of necrotizing enterocolitis in infants [119]. BCFA can also suppress LPS-induced IL-8 expression and reduce the expression of TLR4 in human intestinal epithelial cells [120]. All these data suggest the importance of controlling circulating BCAA levels in prevention of immunometabolic diseases. Further studies are needed to explore the role of other bacterial species in BCAA metabolism, while enhancing BCAA catabolism by modifying gut microbiota population could therefore be beneficial for host metabolism.

### 2.3. Trimethylamine-N-Oxide

Gut microbiota can metabolize dietary L-carnitine, choline, and lecithin into trimethylamine (TMA), which is then converted to trimethylamine-*N*-oxide (TMAO) by flavin-monooxigenenase 3 (FMO3) in the host liver [121,122]. Plasma TMAO concentrations are positively associated with *Prevotella, Peptococcaceae,* and *Clostridiales*, while it is negatively correlated with *Faecalibacterium prausnitzii* [123,124]. TMAO can induce vascular inflammation through MAPK and NF-κB signaling in endothelial cells [125]. Circulating levels of TMAO are positively associated with the risk of atherosclerosis and other cardiovascular diseases [126,127,128]. Excessive TMAO is detected in type 2 diabetic patients with coronary artery disease [129]. Knocking down of host liver FMO3 in low density lipoprotein receptor (*Ldlr*)-deficient mice reduces the hepatic and plasma lipid levels, bile acid pool size, liver triglyceride secretion, ketone bodies, and glucose and insulin levels, resulting in the prevention of atherosclerosis [130]. Moreover, suppression of TMAO generation with a small molecule inhibitor of microbial TMA production can prevent atherosclerosis, suggesting the modification of the gut microbiota to suppress TMAO generation can be a potential target to prevent atherosclerosis and other diseases [131].

Elevated serum TMAO contributes to the pathogenesis of atherosclerosis by interfering with cholesterol metabolism, inducing inflammation, as well as altering the functions of endothelial cells, foam cells and macrophages [132,133]. TMAO increases TLR4 expression in endothelial cells. Inhibition of TLR4 expression protects endothelial cells from TMAO-associated tight junction protein disruption, as well as prevents metabolic inflammation [134]. TMAO impedes reverse cholesterol transport pathway by interrupting bile synthesis and metabolism [135]. Altered cholesterol metabolism is critical for the pro-inflammatory responses and pathogenesis of atherosclerosis, while accumulation of cholesterol accelerates pro-inflammatory responses [136]. TMAO can cause changes in the entire macrophage reverse cholesterol transport pathway [132], and mice fed with TMAO-containing diet show reduced rate of macrophage-specific reverse cholesterol transport [123]. TMAO can promote macrophage cholesterol accumulation in a microbiota-dependent manner by increasing cell surface expression of CD36 and scavenger receptor A (SR-A1) [137].

Plasma proteins associated with inflammation, cardiometabolic disease, and kidney disease also correlate with TMAO. Increased TMAO-producing bacteria are found in patients with rheumatoid arthritis, a chronic inflammatory disease which shares similar pathogenesis to atherosclerosis [133,138]. Elevated TMAO level is also detected in the animal model of arthritis [139]. TMAO enhances M1 macrophage polarization and activates Th1 and Th17 reactions via NLRP3 inflammasome activation [140], as well as promoting the activity of caspase-1, and the production of IL-1β and IL-18 [141,142]. TMAO modulates the activation of aortic macrophages and upregulates the gene expression of IL-1β in atherosclerotic plaque [143]. Recently, TMAO has also been associated with increased proinflammatory CD14^++^CD16^+^ monocytes in stroke patients [144]. In addition, TMAO can alter bile acid metabolism, partly by reducing bile acid synthesis and liver bile acid transporters to decrease the bile acid pool [123]. These suggest TMAO as an important microbial modulator of host immunometabolism.

### 2.4. Secondary Bile Acids

Bile acids are important digestive surfactants that facilitate digestion and absorption of lipids and fat-soluble vitamins. Primary bile acids are endogenously synthesized from cholesterol in liver, which are then further metabolized by the gut microbiota into secondary bile acids. These microbial-derived bile acids contribute to the host bile acid pool, while significant alterations of bile acid pool in germ-free mice compared to conventional mice are reported [145,146]. Moreover, bariatric surgery has been shown to associate with changes in the bile acids metabolism and in the microbiota population [147]. Moreover, fecal transplantation from patients that had Roux-en-Y gastric bypass to germ-free mice leads to less fat gain and increased postprandial bile acid compared to those receiving microbiota from obese people [148], suggesting the interrelationship between host energy metabolism and gut microbiota.

Secondary bile acids are important microbial metabolites that can activate important receptors [such as FXR and G-protein-coupled bile acid receptor 1 (GPBAR-1)] in the host tissues in order to regulate metabolic and immune processes [149]. Gut microbiota affects the bile acid pool not only by producing secondary bile acid but also inhibits host hepatic bile acid synthesis by inhibiting FXR [145]. It has been shown that bile acids have direct antimicrobial effects by destroying bacterial membranes due to their detergent properties, as well as indirect effects through activating FXR and inducing the expression of inducible nitric oxide synthase (iNOS) and IL-18 that modulate the gut microbiota via the immune system [149]. In addition, recent studies have demonstrated that the activation of intestinal FXR leads to hyperglycemia in obese state, suggesting that inhibition of FXR signaling might be efficient for preventing hyperglycemia [150,151]. Long-term oral treatment with FXR agonist results in exacerbated weight gain and glucose intolerance in obese mice [152]. *Fxr*-deficient mice on HFD feeding or cross-bred on a *ob*/*ob* background show protection against diet-induced obesity and improvement of glucose homeostasis compared to control mice [153]. Moreover, double *Fxr* and *Ldlr*-deficient mice on high-fat diet have improved lipid profile and ameliorated diet-induced obesity and atherosclerosis [154]. 

The receptor of secondary bile acids, GPBAR-1, has a role in energy homeostasis by promoting intracellular thyroid hormone activity as well as promoting thermogenesis in adipose tissues [155]. GPBAR-1-specific agonist INT-777, which is a derivative of chenodeoxycholic acid, ameliorates hepatic steatosis, adiposity, and improves insulin sensitivity in HFD-fed mice [156]. GPBAR-1 signaling controls glucose homeostasis by stimulating energy expenditure in brown adipose tissues and muscles and by promoting the release of GLP-1 in intestinal L cells [156]. Interestingly, a recent study has shown that FXR is also expressed in intestinal L cells and regulates GLP-1 synthesis [157].

Chronic exposure to high levels of bile acid can induce inflammation [158]. Since chronic hyperglycemia and oxidative stress are closely related to changes in monocyte and macrophage functions [159], modulation of GPBAR-1/FXR signaling by the gut microbiota may indirectly affect host immune functions. Moreover, Macrophages are major regulators of cytokine production in the gastrointestinal tract, while macrophages are activated by the binding of secondary bile acids with GPBAR-1 [160]. GPBAR-1 activation induces a partial transformation of macrophages from M1 to M2 phenotype, leading to an elevated IL-10 level which inhibits pro-inflammatory cytokines (TNF-α and IL-6) [161]. In this aspect, similar to TLR4, GPBAR-1 is also critical to recognize pathogen-associated molecular patterns and to activate immunity [162]. A recent study has shown that activation of GPBAR-1 significantly inhibits the TLR4/NF-κB pathway leading to the reduction of liver inflammation [163]. However, other studies have suggested that pharmacological activation of FXR ameliorates inflammation and preserves the intestinal barrier integrity in two murine colitis models [164], which is also mediated by the NF-κB signaling pathway [165]. This controversy of FXR effects on inflammatory response needs to be clarified in further studies.

### 2.5. Tryptophan Microbial Metabolites and Others

There is an array of bioactive microbial metabolites derived from the essential aromatic amino acid tryptophan. A few commensal bacteria are identified, including *Peptostreptococcus russellii*, *Clostridium sporogenes*, and *Lactobacillus* spp, to metabolized tryptophan into indole and its derivatives [166,167,168]. Many tryptophan derivatives, such as indole-3-acid-acetic (IAA), indole-3-aldehyde (IAld), indole-3-acetaldehyde (IAAld), indole-3-propionic acid (IPA), and indoleacrylic acid are ligands for aryl hydrocarbon receptor (AhR) [169,170], which is a transcription factor that plays an important role in immunological response and inhibits inflammation [171]. AHR signaling contributes to immune homeostasis by modulating T cell differentiation, Th17 development, and IL-22 production [172,173,174].

Recently, epidemiologic studies have shown the association between IPA and metabolic diseases. Serum IPA levels are negatively correlated with the risk of type 2 diabetes and low-grade inflammation [175,176]. In addition, administration of IPA represses hepatic inflammation and liver injury in rats, via inhibiting NF-κB signaling and reducing the levels of proinflammatory cytokines (TNFα, IL-1β, and IL-6) in response to endotoxin in macrophages [177]. Moreover, administration of IPA inhibits the expression of genes promoting fibrosis, and attenuates diet-induced anti-non-alcoholic steatohepatitis (NASH) phenotypes in HFD-fed rats [177]. Moreover, administration of IPA significantly ameliorates colitis and induces IL-10-mediated anti-inflammatory pathway *in vitro* [178].

Imidazole propionate has been shown to associate with glucose intolerance, deteriorated insulin signaling and diabetes [179]. Imidazole propionate impairs insulin signaling by activating MAPK, which then promotes p62 phosphorylation and mTORC1 activation [179]. Indoxylsulphate induces TNF-α production in macrophages in an AhR- and NF-κB-dependent manner [180]. 

Apart from those mentioned above, gut microbiota produce a broad spectrum of epigenetically active metabolites, such as folate and vitamins, that regulate the activity of host chromatin-modulating enzymes and genetic responses to environmental signals [181]. However, the function of these microbial metabolites in the host immunometabolism is still not elucidated. Nevertheless, the complicated regulation of the host immunometabolism by the gut microbiota and its metabolites warrant further investigations.

## 3. Metabolism of Polyphenols by Gut Microbiota

Dietary polyphenols are perceived as xenobiotics in humans, and their biological availability is reasonably poor comparing to other micro- and macro-nutrients. The structural complexity and polymerization of polyphenols also affects their absorption in small intestines [182]. Absorption of the consumed polyphenols in the small intestines is particularly low (less than 10%). The poor bioavailability is, therefore, a major concern for their development as therapeutic agents [183]. The unabsorbed polyphenols accumulate in the large intestines along with the bile conjugates in the lumen and are exposed to the gut microbial enzymatic activities [6].

In humans, the metabolism of dietary polyphenols is an important aspect that deserves detailed consideration and warrants further studies. Gut microbiota significantly contributes to the metabolism of dietary polyphenols leading to the generation of *de novo* and potentially bioactive compounds. As mentioned, more than 90% of the dietary polyphenols are not absorbed in the small intestines and reach the large intestines; thus gut microbiota is critically important in turning these polyphenols into bioavailable products [184]. In general, gut microbiota metabolizes glycosylated polyphenols into lower molecular weight phenolic compounds, such as small phenolic acids [185]. Indeed, these gut microbiota-derived polyphenolic metabolites are also important essential bioavailable polyphenolic acids. Polyphenols have been shown to undergo various enzymatic processes by gut microbiota, through which the polyphenol derivatives are in a form capable of being absorbed or even more bioactive [186,187]. Therefore, the protective effects of polyphenols also depend on how gut microbiota metabolize these compounds.

Currently, there are mainly *in vitro* studies and a few *in vivo* studies focusing on the effect of microbiota-derived polyphenolic metabolites in immunometabolism (Table 1). Gut microbiota are responsible for the metabolism of resveratrol to piceid [188], which shows a higher bioavailability and antioxidant activity than resveratrol [189]. One of the derivatives from proanthocyanidins, 3-Hydroxyphenylpyruvic acid (3-HPPA), has been shown to attenuate oxidized-LDL-induced cellular oxidative stress and inflammation via NF-κB pathways and inhibit the conversion of macrophage into foam cells via regulating cellular lipid metabolism *in vitro* [190]. On the other hand, 3-(3′-hydroxyphenyl) propionic acid (3-HPP) has been shown to regulate Akt and eNOS via insulin-stimulated signaling and promote NO production during high glucose conditions in endothelial cells *in vitro* [191]. Microbiota-derived metabolite of anthocyanins, protocatechuic acid (PCA), possesses anti-inflammatory effects by regulating NF-kB and MAPK activation *in vitro* [192] and attenuates oxidative stress and apoptosis by reducing expressions of TNF-a, IL-1β, and IL-6 *in vivo* [193]. Urolithins, a derivative of ellagitannins, has been shown to reduce TNFα-induced inflammation through inhibiting histone acetyltransferase (HAT) activity in monocytes *in vitro* [194]. A large portion of dietary anthocyanins are degraded by gut microbiota to free anthocyanidins, protocatechuic acid (PCA) and gallic acid (GA) [195], which confer protective effects against obesity and insulin resistance [196]. Moreover, GA has been shown to regulate mitochondrial function by activating AMPK and PGC1α, while SIRT1 knockdown significantly blunted such effect [197]. In HFD-fed mice, GA treatment protects diet-induced obesity, improves glucose and insulin homeostasis, and promotes thermogenesis by increasing uncoupling protein 1 (UCP1) expression in brown adipose tissue [197]. The main soy isoflavone, daidzein, can be metabolized by the gut microbiota to equol [198], which shows higher anti-oestrogenic activity, antioxidant capacity, and anti-cancer effects than daidzein [199]. Equol has been shown to reduce triglycerides, total cholesterol, and LDL-cholesterol and increase HDL-cholesterol in HFD-fed mice [200]. A study of the Japanese population suggests the potential role of equol in glycemic control, while overweight or obese group have more equol non-producers than the normal weight group [201]. These studies suggest that gut microbiota is particularly important in the metabolism of polyphenols into essential bioactive compounds. Nevertheless, the functions of these polyphenolic metabolites are not thoroughly studied. Further *in vivo* and clinical studies are warranted to investigate the function of these metabolites in modulating the host immunometabolism. 

## 4. Effect of Polyphenols on the Composition of Gut Microbiota and Host Metabolism

Growing studies support the hypothesis that polyphenols with poor bioavailability possibly act primarily through the gut microbiota remodeling [21]. Unabsorbed polyphenols that reach the colon, as well as the metabolites generated, may interact with the gut microbiota, and may modulate microbial composition and function [207,208]. Previous polyphenol researches focused on their antimicrobial activity, while the concept of polyphenols as potential prebiotics to shape the gut microbiota composition is emerging [22]. In general, lowering the *Firmicutes/Bacteroidetes* ratio and colonization of specific beneficial bacterial species are considered to provide protection to the host against certain pathologies [20]. Gram-positive bacteria are more prompted to the action of polyphenols than Gram-negative bacteria, possibly due to the differences in their wall composition [6]. Polyphenols have been shown to increase the population of beneficial species such as *Bifidobacterium* and *Lactobacillus,* which contribute to the gut barrier protection; *Faecalibacterium prausnitzii,* which possess anti-inflammatory effect by inhibiting the activation of NF-kB; and *Roseburia* sp., which produces butyrate [212,213]. Recent studies have suggested that the mucin-degrading bacterium *Akkermansia muciniphila* is an important player contributing to the anti-inflammatory and beneficial effects of dietary polyphenols in obesity [209,210,211]. *Akkermansia muciniphila* is presented in great abundance in healthy subjects, but it is reduced in patients with inflammatory and gastrointestinal diseases, obesity, and diabetes [210]. Moreover, ellagitannins have been shown to stimulate the growth of *Akkermansia muciniphila in vivo* [211]. Although the effect of polyphenols on gut microbiota modulation has gained much attention in recent years, the effects on specific gut bacteria and metabolites production are still not clear. Here, we summarize recent publications on the effect of polyphenols in modulation of gut microbiota and immunometabolism in animal models (Table 2).

Resveratrol treatment (0.4% in diet) has been shown to enhance the population of *Akkermansia muciniphila*, promote bile acid synthesis and reduce serum TMA and TMAO levels in both wild type mice and ApoE-/-mice [135]. However, another study has shown that resveratrol (15 mg/kg/day) normalizes serum insulin levels and insulin resistance without significant changes in *Firmicutes/Bacteroidetes* ratio in high-fat-and-sucrose-diet (HFSD)-fed Wistar rats [214]. Camu camu extract (containing around 30% proanthocyanidins 30%) has been shown to prevent high-fat-high-sucrose-diet (HFHSD)-induced obesity, partly by increasing *Akkermansia muciniphila* population and the proportion of secondary and unconjugated bile acids in the plasma of mice [219]. Interestingly, instant caffeinated coffee has been shown to prevent HFD-induced obesity, partly by reducing *Firmicutes/Bacteroidetes* ratio and serum BCAA level, while increasing serum SCFA level in rat [228]. Moreover, purified citrus polymethoxyflavone-rich extract can attenuate HFD-induced obesity by reducing *Firmicutes/Bacteroidetes* ratio and downregulating mTOR signaling [103]. Two plant-derived polyphenols, carnosol and curcumin, have been shown to prevent the increase in glycolysis and spare respiratory capacity in response to LPS stimulation in human dendritic cells [18]. The regulation of metabolism by the two polyphenols is via activation of AMPK, which results in the inhibition of mTOR signaling [18]. Carnosol and curcumin also upregulate heme oxygenase-1 (HO-1), which is an important anti-inflammatory and antioxidant enzyme [232]. Recently, an untargeted metabolomics study has revealed the induction of anti-inflammatory prostaglandin pathway and modulation of gut microbiota by the consumption of curcumin-containing *curcuma longa* L. extract [233]. 

The discrepancies between the results of different studies could be due to the low bioavailability of polyphenols. Most studies are reported without considering or measuring the bioavailability and the chemistry of polyphenols in the animals. Moreover, another limitation is that the results obtained from *in vitro* studies (summarized in [234]) and *in vitro* animal studies about the role of polyphenol on gut microbiota cannot be directly extrapolated to physiological context, due to the very high doses polyphenols used. The wide variation in response to polyphenols could be due to the inter-individual differences in genome and microbiota on the studied models. Human intervention studies may further provide a better model for studying the effect of polyphenol on gut microbiota and immunometabolism modulation. To date, only a few human intervention studies have investigated the *in vivo* impact of polyphenols on the gut microbiota [235,236,237,238]. However*, in vivo* human studies also hold inevitable limitations including applied microbial techniques. Further *in vivo* researches are needed to understand the effect of polyphenols on gut microbiota and immunometabolism. Measuring the changes in microbial metabolome, together with the bacterial population, may act as a better study strategy.

## 5. Future Direction and Summary

Gut microbiota significantly contributes to the host immunometabolism. Gut microbiota is also an important factor that contributes to the bioavailability and effects of dietary polyphenols. Polyphenols are known as antioxidant and anti-inflammatory molecules. In addition, the induced changes of gut microbiota and the gut microbiota-derived polyphenolic metabolites also significantly contribute to the health effects of polyphenols in immunometabolism. The beneficial effects of polyphenols on immunometabolism are highly dependent on the gut microbiota. This review highlights the contribution of gut microbiota and its metabolites in host immunometabolism, as well as the effect of polyphenols in animal models of immunometabolic disease. However, the observed phenotypes in these animal studies are sometimes controversial and are not always conserved in human studies. The use of antibiotics treatment may provide inaccurate results as some resistant species may still be presented or selected [47]. It is important to note that 85% of the murine microbiome species are not yet detected in human microbiomes [239]. The use of humanized animal may be a good model to investigate the interaction between microbiota and polyphenols in immunometabolic diseases [48]. However, the humanization of mouse models may not adequately display the whole spectrum of relevant phenotype of human diseases [45,46]. Antibiotics-treated mice model can also be considered [47]. These challenges in screening the accurate changes in the population of gut microbiota may be overcome by targeting the microbial metabolome. Currently, strong evidence on a causal relationship between the microbial metabolome and effect of polyphenols in modulating immunometabolism is still missing. To identify novel potential treatment targets, future study direction may focus on the microbial metabolomics and their effects on host immunometabolism.

## Figures and Tables

**Table 1:** Microbial-derived polyphenolic metabolites and their protective effects.

Polyphenol	Metabolizing Bacteria	Main Metabolite	Effects in Immunometabolism	Refs
Proanthocyanidins	*Lactobacillus plantarum*	3-Hydroxyphenylpyruvic acid (3-HPPA)	↓ macrophage foam cell formation ↓ oxidative stress ↓ inflammation	[190]
	*Enterococcus casseliflavus, Clostridium coccoides,* and *C. orbiscindens*	3-(3′-hydroxyphenyl) propionic acid (3-HPP)	↑ NO production ↓ hypertension	[191,202,203]
	*Lactobacillus plantarum, Lactobacillus casei, Lactobacillus acidophilus,* and *Bifidobacterium lactis*	3-hydroxybenzoic acid (3-HBA)	↓ inflammation	[186,204]
Anthocyanins	*Lactobacillus* spp. and *Bifidobacterium* spp.	Protocatechuic acid (PCA)	↓ obesity ↓ monocyte adhesion molecules ↓ atherogenesis by ↓ monocyte infiltration ↓ inflammation	[192,193,196]
	*Lactobacillus plantarum 299v* and *Bacillus subtilis*	Gallic acid (GA)	↑ thermogenesis ↓ obesity ↓ inflammatory arthritis, ↓ IL-2, IFN-γ, TNF-α, IL-4 & IL-5 ↓ LPS-induced inflammation	[197,205,206,207]
Daidzein	*Bacteroides Ovatus* spp., *Strepotococcus intermedius* spp., and *Ruminococcus productus* spp.	Equol	↓ atherosclerotic lesions ↓ IL-12/IL-18 induced NK cell IFN-γ production ↓ oxidative stress	[198,200,208]
Resveratrol	*Bacillus cereus*	Piceid	↑ bioavailability than resveratrol ↓ LPS-induced endothelial barrier disruption	[189,204]
Ellagitannins	*Akkermansia muciniphila, Butyrivibio* spp., *Gordonibacter urolithinfaciens, Gordonibacter pamelaeae,*	Urolithins	↑ AMPK activity ↓ TNFα-induced inflammation ↓ obesity	[194,209,210,211]
Lignans	*Bacteroides distasonis, Bacteroides fragilis, Bacteroides ovatus, Clostridium cocleatum, Clostridium saccharogumia,*	Enterolactone	↓ oxidative stress Unknown in immunometabolism yet	[205,206]

IL, Interleukin; IFN, interferon; TNF, tumor necrosis factor; NK, natural killer; LPS, lipopolysaccharides; AMPK, 5′ adenosine monophosphate-activated protein kinase.

**Table 2:** Effect of polyphenols in different animal models of metabolic complications.

Polyphenol	Dose	Animal Model	Changes in Microbiota	Metabolic or Functional Effects	Refs
Resveratrol	0.4% in diet	WT C57BL/6J mice	↑ *Bacteroides*, *Lactobacillus*, *Bifidobacterium*, and *Akkermansia*; ↓ *Prevotella*, *Ruminococcaceae*, *Anaerotruncus*, *Alistipes*, *Helicobacter*, and *Peptococcaceae*; ↑ *Bacteroidetes* (35.1% to 44.1%), ↓ *Firmicutes* (50.3% to 35.4%).	↑ bile acid deconjugation;↑ hepatic bile acid synthesis; ↓ plasma TMA and TMAO levels.	[135]
	0.4% in diet	Choline-treated ApoE-/-mice	↑ *Bacteroides*, *Lactobacillus*, *Bifidobacterium*, and *Akkermansia*; ↓ *Prevotella*, *Ruminococcaceae* and *Biophila*; ↑ *Bacteroidetes* (20.6% to 34.0%), ↓ *Firmicutes* (60.1% to 50.1%).	↑ bile acid deconjugation; ↑ hepatic bile acid synthesis; ↓ atherosclerosis; ↓ plasma TMA and TMAO levels.	[135]
	15 mg/kg/day	HFSD-fed Wistar rats	No change in *Bacteroidetes* and *Firmicutes*	↓ serum insulin levels and insulin resistance	[214]
	200 mg/kg/day	HFD-fed Kunming mice	↑ *Lactobacillus* and *Bifidobacterium;* ↓ *Enterococcus faecalis*; ↑ *Bacteroidetes*/*Firmicutes* ratio;	↑ *Fiaf* expression in intestine; ↓ body and visceral fat weight; ↓ blood glucose and lipid levels.	[215]
	1 mg/kg/day	DSS-induced colitis Fischer F344 rats	↑ *Bifidobacterium*, *Lactobacilli*; ↓ *Enterobacteria*	↑ colonic mucosa architecture; ↓ body weight loss; ↓ inflammation	[216]
Piceatannol (resveratrol analogue)	0.25% in diet	HFD-fed C57BL/6 mice	↑ *Lactobacillus*, *Firmicutes* (45.8 to 74.5%); ↓ *Bacteroidetes* (52.0 to 21.4%).	↓ body weight and adiposity; ↓ blood glucose level; ↓ serum LDL-C, HDL-C and the LDL-C/HDL-C ratio.	[217]
	45 mg/kg/day	Obese Zucker rats	Slight changes in *Bacteroidetes* and *Firmicutes*	No profound effects	[218]
Camu camu extract (proanthocyanidins 30%)	200 mg/kg/day	HFHSD-fed C57BL/6J mice	↑ *Akkermansia muciniphila*, *Bifidobacterium* and *Barnesiella* ↓ *Lactobacillus.*	↑ glucose tolerance and insulin sensitivity; ↑ energy expenditure; ↓ body weight gain and fat accumulation; ↓ adipose tissue inflammation and metabolic endotoxemia;↓ hepatic steatosis; alter plasma bile acid pool size and composition.	[219]
Pomegranate peel extract (containing 30% polyphenol, 8% punicalagin and 5% ellagic acid)	0.2% in water (6 mg/day)	HFD-fed Balb/c mice	↑ *Bifidobacterium *spp., *Lactobacillus *spp., *Bacteroides–Prevotella *spp.	↓ serum cholesterol levels; ↓ inflammatory markers expression in visceral fat.	[220]
Quercetin	30 mg/kg/day	HFSD-fed Wistar rats	↓ *Erysipelotrichaceae*, *Bacillus*, *Eubacterium cylindroides*; ↓ *Firmicutes*/*Bacteroidetes* ratio;	↓ serum insulin levels and insulin resistance; ↓ microbiota dysbiosis.	[214]
Quercetin and Resveratrol	30 mg/kg/day, 15 mg/kg/day, respectively	HFD-fed Wistar rats	↑ *Bacteroidales*_S24-7_group, *Christensenellaceae*, *Akkermansia*, *Ruminococcaceae* and its genera *Ruminococcaceae*_UCG-014, and *Ruminococcaceae*_UCG-005; ↓ *Desulfovibrionaceae*, *Acidaminococcaceae*, *Coriobacteriaceae*, *Bilophila*, *Lachnospiraceae* and its genus *Lachnoclostridium*; ↓ *Firmicutes* ↓ *Firmicutes*/*Bacteroidetes* ratio	↑ adiponectin; ↓ body weight gain and visceral fat weight; ↓ serum lipids; ↓ serum inflammatory markers (TNF-α, IL-6, MCP-1);	[221]
Daidzein	20mg/kg/day	B6C3F1 mice	Not specified	↑ T cell population; ↓ B cell population; ↓ % of late apoptotic thymocytes.	[222]
	0.1% in diet	db/db mice	Not specified	↑ AMPK phosphorylation; ↓ fasting blood glucose, serum total cholesterol levels.	[223]
Polymeric procyanidins	0.5% in diet	HFHS-fed C57BL/6J mice	↑ *Akkermansia*; ↓ *Clostridium*, *Lachnospiraceae*, and *Bifidobacterium*; ↓ *Firmicutes*/*Bacteroidetes* ratio;	↑ lipid metabolism; ↓ weight gain; ↓ circulating LPS and gut permeability;	[224]
Powered green tea leaves (*Camellia sinensis*)	4% in diet (in combination with *Lactobacillus plantarum*)	HFD-fed C57BL/6J mice	↑ *Akkermansia;* ↑ *Lactobacillus.*	↓ body fat and hepatic triacylglycerol and cholesterol accumulation; ↓ inflammation.	[225]
Canarium album extract (containing around 465.35 mg/g polyphenol)	20 mg/kg/day	HFD-fed Kunming mice	↑ *Firmicutes* and *Verrucomicrobia*; ↑ *Akkermansia;* ↓ *Bacteroidetes.*	Not specified	[226]
Concord grape polyphenols	1% in diet	HFD-fed C57BL/6J mice	↑ *Akkermansia muciniphila* ↓ *Firmicutes*/*Bacteroidetes* ratio	↓ weight gain, adiposity and serum inflammatory markers; ↓ glucose intolerance.	[227]
Coffee (instant caffeinated coffee)	20 g/L in water	HFD-fed SD rats	↑ *Enterobacteria;* ↓ *Clostridium* Cluster XI; ↓ *Firmicutes*/*Bacteroidetes* ratio	↑ serum SCFA level ↓ body weight, adiposity, liver triglycerides and energy intake; ↓ insulin resistance; ↓ serum BCAA level.	[228]
Five types of arctic berries powdered extract bog blueberry, cloudberry, crowberry, alpine bearberry, lingonberry	200 mg/kg/day	HFHS-fed C57BL/6J mice	↑ *Akkermansia muciniphila*, *Turicibacter*, *Oscillibacter*.	↓ fasting and postprandial hyperinsulinemia; ↓ liver triacylglycerol deposition; ↓ circulating endotoxins; ↓ hepatic and intestinal inflammation.	[229]
Plum juice (containing around 1,270 mg gallic acid equivalents/mL)	drinking water	Obese Zucker rats	↑ *Lactobacillus* and members of *Ruminococcacea*.	↓ body weight; ↓ fecal acetic and propionic acid level	[230]
Purified citrus polymethoxyflavone-rich extract (including 38.51% (w/w) nobiletin, 15.62% tangeretin, 3.43% sinensetin, and 3.29% 3,5,6,7,8,3′,4′-heptamethoxyflavone)	120 mg/kg/day	HFD-fed C57BL/6J mice	↑ *Bacteroides ovatus*; ↓ *Firmicutes*/*Bacteroidetes* ratio.	↑ serum HDL-C; ↓ body weight gain, serum TC, TG and LDL-C; ↓ inflammation; ↓ gut dysbiosis; ↓ mTOR/P70S6K/SREBP pathway	[103]
Red raspberry polyphenols from whole fruit, seed, and pulp	whole fruit (0.4% in diet); seed (0.1% in diet); pulp (0.3% in diet)	HFD-fed C57BL/6 mice	Not specified	↑ energy expenditure; ↓ body weight gain, dyslipidemia, and insulin resistance ↓ inflammation, macrophage recruitment, ↓ adipocyte size in epididymal fat ↓ NLRP3 inflammasome activation	[231]

WT, wild type; TMA, trimethylamine; TMAO, trimethylamine-N-oxide; apoE, Apolipoprotein E; HFSD, high-fat-and-sucrose-diet; HFD, high-fat diet; DSS, dextran sulfate sodium; LDL-C, low-density lipoprotein-cholesterol; HDL-C, high-density lipoprotein-cholesterol; HFHSD, high-fat-high-sucrose-diet; TNF, tumor necrosis factor; IL, Interleukin; MCP, monocyte chemoattractant protein; LPS, lipopolysaccharides; SCFA, short-chain fatty acids; BCAA, branched-chain amino acids; TC, total cholesterol; TG, triglyceride; mTOR, mammalian target of rapamycin; P70S6K, p70S6 kinase; SREBP, sterol regulatory element-binding protein; NLRP, NACHT, LRR and PYD domains-containing protein.
